# Targeted Sequencing of Candidate Regions Associated with Sagittal and Metopic Nonsyndromic Craniosynostosis

**DOI:** 10.3390/genes13050816

**Published:** 2022-05-03

**Authors:** Cristina M. Justice, Anthony M. Musolf, Araceli Cuellar, Wanda Lattanzi, Emil Simeonov, Radka Kaneva, Justin Paschall, Michael Cunningham, Andrew O. M. Wilkie, Alexander F. Wilson, Paul A. Romitti, Simeon A. Boyadjiev

**Affiliations:** 1 Genometrics Section, Computational and Statistical Genomics Branch, National Human Genome Research Institute, National Institute of Health (NIH), Baltimore, MD 21224, USA; cmj@mail.nih.gov (C.M.J.); afw@mail.nih.gov (A.F.W.); 2 Statistical Genetics Section, Computational and Statistical Genomics Branch, National Human Genome Research Institute, National Institute of Health (NIH), Baltimore, MD 21224, USA; anthony.musolf@nih.gov; 3 Department of Pediatrics, University of California Davis, Sacramento, CA 95616, USA; acuellar2001@gmail.com; 4 Fondazione Policlinico Universitario A. Gemelli IRCCS, 00168 Rome, Italy; wanda.lattanzi@unicatt.it; 5 Department of Life Sciences and Public Health, Università Cattolica del Sacro Cuore, 00168 Rome, Italy; 6 Pediatric Clinic, Alexandrovska University Hospital, Medical University of Sofia, 1431 Sofia, Bulgaria; simeonov.emilg@gmail.com; 7 Molecular Medicine Center, Department of Medical Chemistry and Biochemistry, Medical Faculty, Medical University of Sofia, 1431 Sofia, Bulgaria; kaneva64@yahoo.com; 8 Bioinformatics Core, Computational and Statistical Genomics Branch, National Human Genome Research Institute, National Institute of Health (NIH), Bethesda, MD 20892, USA; justin.paschall@nih.gov; 9 Seattle Children’s Craniofacial Center, Center of Developmental Biology and Regenerative Medicine and Division of Craniofacial Medicine, Department of Pediatrics, University of Washington, Seattle, WA 98105, USA; michael.cunningham@seattlechildrens.org; 10 MRC Weatherall Institute of Molecular Medicine, University of Oxford, John Radcliffe Hospital, Oxford OX3 9DS, UK; andrew.wilkie@imm.ox.ac.uk; 11 Oxford Centre for Genomic Medicine, Oxford University Hospitals NHS Foundation Trust, Oxford OX3 9DS, UK; 12 Craniofacial Unit, Oxford University Hospitals NHS Foundation Trust, Oxford OX3 9DS, UK; 13 Department of Epidemiology, College of Public Health, The University of Iowa, Iowa City, IA 52242, USA

**Keywords:** craniosynostosis, metopic, sagittal, nonsyndromic  non-syndromic, targeted sequencing, *BMP7*, *BMP2*, *BBS9*, *BMPER*

## Abstract

Craniosynostosis (CS) is a major birth defect in which one or more skull sutures fuse prematurely. We previously performed a genome-wide association study (GWAS) for sagittal non-syndromic CS (sNCS), identifying associations downstream from *BMP2* on 20p12.3 and intronic to *BBS9* on 7p14.3; analyses of imputed variants in *DLG1* on 3q29 were also genome-wide significant. We followed this work with a GWAS for metopic non-syndromic NCS (mNCS), discovering a significant association intronic to *BMP7* on 20q13.31. In the current study, we sequenced the associated regions on 3q29, 7p14.3, and 20p12.3, including two candidate genes (*BMP2* and *BMPER)* near some of these regions in 83 sNCS child-parent trios, and sequenced regions on 7p14.3 and 20q13.2-q13.32 in 80 mNCS child-parent trios. These child-parent trios were selected from the original GWAS cohorts if the probands carried at least one copy of the top associated GWAS variant (rs1884302 C allele for sNCS; rs6127972 T allele for mNCS). Many of the variants sequenced in these targeted regions are strongly predicted to be within binding sites for transcription factors involved in craniofacial development or bone morphogenesis. Variants enriched in more than one trio and predicted to be damaging to gene function are prioritized for functional studies.

## 1. Introduction

Craniosynostosis (CS) is a major structural birth defect characterized by the premature fusion of one or more cranial sutures and is estimated to have a prevalence of 1:2500 live births [1]. Surgical intervention is usually necessary to prevent intracranial hypertension, which can lead to neurocognitive impairment. Approximately 80% of children with CS present as an isolated phenotype (i.e., nonsyndromic CS [NCS]) [2], where premature suture fusion is the only major defect. NCS phenotypes are classified based on suture involvement (sagittal, coronal, metopic, lambdoid), of which sagittal suture stenosis (45% of NCS cases) is the most common phenotype [3]. Most individuals affected with NCS present sporadically, although approximately 6–8% of families have been reported to have multiple affected individuals (Boyadjiev and International Craniosynostosis Consortium, 2007). Specifically, for midline NCS (sagittal and metopic NCS), 5.7% of individuals affected with sagittal NCS (sNCS) and 6.8% of those with metopic NCS (mNCS) report a family history of the disorder (Lajeunie et al., 2005). Additional evidence of a genetic component is based on discrepant male to female ratios, twin studies, and increased recurrence risks [4,5]. The etiology of NCS represents a large gap in our current knowledge; however, genomic technologies, such as genome-wide association studies (GWASs) and genome sequencing strategies, have begun to narrow this knowledge gap.

Pathogenic variants in a handful of genes (usually showing incomplete penetrance) explain less than 10% of the expression of NCS in all patients. Sequencing studies have reported loss of function pathogenic variants in *SMAD6* in 7% of patients with midline NCS [6], pathogenic variants in *ERF* in five individuals from 12 mixed (sagittal, lambdoidal, coronal, and metopic) NCS multiplex families [7], and pathogenic variants in *TCF12* in 10% of those affected with unilateral coronal NCS (cNCS) and 32% of those affected with bicoronal NCS [8].

We previously conducted the first GWAS of sNCS [9] and the first GWAS of mNCS [10]. Both studies used parent-child trio data and were analyzed using the transmission disequilibrium test (TDT). The purpose of this study is to perform fine mapping of the associated regions identified previously by GWAS to identify both common and rare potentially causal variants. Because the TDT is a test of association in the presence of linkage (Spielman et al., 1993) and tracks the genetic transmission of an allele and trait from parent to child, it is not affected by population stratification and allows us to use a far smaller sample size than population based GWAS, which is ideal for rare diseases, such as craniosynostosis.

Our previous TDT analyses identified genome-wide significant associations to sNSC on chromosome 20p12.3 near *BMP2*, chromosome 7p14.3 within *BBS9* 20p12.3, and chromosome 3q29 near *DLG1* [9] whereas our mNCS TDT identified a genome-wide significant association to chromosome 20 q13.2-13.32 within *BMP7*, and a borderline significant association to 7p14.3 intronic to *BBS9*. Both analyses had been performed using GWAS arrays that did not offer coverage of the whole genome. Therefore, it is likely we identified variants in linkage disequilibrium (LD) with a casual variant not present in the initial GWAS array. Our hypothesis for this study is that by performing fine mapping of the associated regions, it is highly likely that we will identify causal variants in LD with our GWAS associated single nucleotide variants (SNVs). For this study, imputation was not considered for two reasons: (1) imputation is unlikely to identify rare variants or *de novos*; (2) genotypes for variants near the rs1884302 were inaccurate when compared to genotyped variants found in the arrays, which is probably due to a large number of probands with the risk allele at this SNV. Imputation produces allele calls at adjacent markers based on what allele the reference populations had at rs1884302; however, the allele calls assigned by imputation differed many times from the allele calls of the few variants near rs1884302 genotyped in the GWAS. Genotypes not in LD with the risk allele were not similarly affected.

The goal of this study is to identify the true causal variant in our previously identified genome-wide significant regions. Thus, we performed targeted sequencing of the association peaks identified in the prior TDT analyses [9,10] to provide a dense marker map (including rare variants) in these regions—20q12.3, 3q29, and 7p14.3 for sNCS and 20q13.2-13.32 and 7p14.3 for mNCS. Because our objective was a deeper exploration of signals already found to be significant, we selected trios for sequencing, rather than the case and controls from the replication cohorts, which allows for the identification of *de novo* variants as well. Moreover, because we wanted to find causal variants in LD with the risk alleles identified in the original GWASs, we selected those trios in which the proband carried the risk allele (rs1884302 for sNCS and rs6127972 for mNCS).

Because sNCS and mNCS are relatively rare disorders, and trios are harder to collect as compared to single isolated cases, our initial sample sizes in the GWASs were relatively small (130 sNCS trios and 215 mNCS trios). However, in both studies, we were able to identify variants that were genome-wide significant and were able to replicate these in separate case-control cohorts, indicating a strong genetic effect leading to these phenotypes.

## 2. Materials and Methods

### 2.1. Human Subjects

Informed written consent was obtained from all patients and/or their parents. This study was approved by the Institutional Review Boards of the participating institutions. For this study, we selected non-Hispanic White (NHW) trios for each phenotype, sNCS and mNCS, from the original GWAS discovery samples. NHW trios were defined as such using principal component analysis, as previously described (Justice et al., 2012; Justice et al., 2020). Trios were preferentially selected if the proband carried at least one copy of the most significant variant identified in each GWAS (rs1884302 C allele for sNCS; rs6127972 T allele for mNCS). DNA was extracted from whole blood or buccal specimens. For the final 83 sNCS trios retained after quality control (QC), 38 were C/T and 45 were C/C at rs1884302. For the 80 mNCS retained after QC, 46 were T/G and 34 were T/T at rs6127972.

### 2.2. Targeted Next Generation Sequencing

The associated regions sequenced for sNCS trios (GRCh37/hg19) were chromosome 3:196,750,207–197,026,927, chromosome 7:33,152,668–34,195,484 (includes *BBS9*, *BMPER*, and the noncoding region between these two genes), along with chromosome 20:6,748,745–6,760,910 (includes *BMP2*) and 20:7065143–7202442 (includes rs1884302), for a total of 1,469,000 bp sequenced. Specimens were sequenced at the NIH Intramural Sequencing Center. The targeted regions of interest (Table S1) were enriched with a custom Nimblegen EZ Choice Enrichment Kit and sequenced on an Illumina HiSeq2500 (2 × 126, v4 chemistry). The sequence reads were aligned with NovoAlign and duplicate read pairs were removed prior to variant calling. Our sNCS trios previously aligned to hg18 were realigned to GRCh37/hg19 using Novocraft 4.02.02 to correspond with the mNCS map.

For the mNCS trios, two 2 Mb target regions (Table S1) encompassing associated variants were sequenced for mNCS trios (GRCh37/hg19):chromosome 20:54,797,057–56,797,057 for rs6127972 (includes *BMP7*) and chromosome 7:32,652,668–34,695,364 for rs10262453 (includes *BBS9* and *BMPER*), for a total of 4,042,816 bp sequenced. Although rs10262453 was only borderline genome-wide significant in the mNCS GWAS [10], it was of particular interest because it acts in opposite directions in mNCS and sNCS, and is conserved across species with a GERP score of 2.39 [11]. Specimens were sequenced at the Center for Inherited Disease Research. Amplified libraries were enriched following the Twist protocol (24 h hybridization) and libraries were sequenced on the NovaSeq 6000 platform using NovaSeq 6000 S1 Reagent Kit.

### 2.3. Allele Calling

The Genome Analysis Toolkit (GATK) best practices [12] pipeline was performed using GATK version 4.1 and included: duplicates marking, base quality score recalibration, gVCF generation using HaplotypeCaller, and joint genotyping of the single sample gVCFs together with GenotypeGVCFs to produce a multisample VCF file. Multisample VCF files for sNCS and mNCS were produced separately, as they mostly involved different regions, and were analyzed separately because they involve different suture phenotypes.

Variants were filtered using VQSR (GATK v4.1). The VCF files produced were analyzed using SVS Golden Helix Software (Bozeman, MT, USA). Variants were dropped prior to analysis if the read depth was <15 or the genotype quality was <20. Variants were also filtered on the expected alternate allele ratio for each genotype, by removing homozygous reference variants with alternate allele ratio ≥0.15, homozygous alternate variants with an alternate allele ratio ≤0.85, and heterozygous variants with an alternate allele ratio <0.3 or >0.7. Only variants in the targeted region were considered for analysis. Multiallelic alleles were split into two by SVS Golden Helix Software. This resulted in 11,794 variants available for sNCS and 34,268 available for mNCS. Variants were also dropped if the missing rate was >20% or were monomorphic. Additionally, after running *de novo* variant analysis, variants with two or more Mendelian errors were dropped, resulting in 5496 and 25,301 variants for sNCS and mNCS, respectively.

Some specimens were dropped prior to analysis due to sample contamination or were part of a trio in which one family member failed to genotype. This reduced the number of individuals available for analysis to 249 (83 trios) for sNCS and 240 (80 trios) for mNCS. The ratio of males to females was 5.4 to 1 in sNCS and 4 to 1 in mNCS.

### 2.4. De Novo Variant Analysis

To identify *de novo* variants, we used a more stringent missing rate (variants dropped if missing rate >2%) and performed Mendelian error analysis using PLINK v1.9 [13]. After performing *de novo* analysis and before running any association analyses, variants with two or more Mendelian inconsistencies were dropped, whereas variants with one Mendelian inconsistency were zeroed out for the trio involved.

### 2.5. Transmission Disequilibrium Test Analysis

Associations between a phenotype (sNCS or mNCS) and each variant were measured using TDT in PLINKv1.9. Because we investigated three signals (on chromosomes 3, 7, 20) for sNCS and two signals (on chromosomes 7 and 20) for mNCS, there was the possibility for locus heterogeneity in these signals (i.e., different trios were driving the different signals on each chromosome). Thus, we ran a leave-one-out analysis (jackknife procedure) where the TDT was run repeatedly with a single trio removed to determine which trios contributed to which signal. The TDT analysis was run a final time on the groups of trios driving each signal. Regional association plots were created using LocusZoom (locuszoom.org). Given that we performed targeted sequencing and had a reduced number of variants compared to a GWAS, we used a significance threshold of *p* < 1 × 10^−5^.

### 2.6. Variant Annotation

We annotated all variants using wANNOVAR [14]. We were particularly interested in potential rare variants in coding regions, with rare being defined as a minor allele frequency (MAF) < 1% in the gnomAD non-Finnish European database (https://gnomad.broadinstitute.org, accessed on 14 February 2022), as these variants would not be analyzed in our TDT analyses. All noncoding variants were annotated using RegulomeDB (https://regulomedb.org, accessed on 14 February 2022) to determine potential transcription factor binding sites (TFBSs).

### 2.7. Rare Variant Analysis

We used gene-based tests to determine if rare variants were, in part, responsible for any of the disease risks in sNCS or mNCS. These variants are underpowered in a traditional single variant TDT analysis; thus, we used collapsing methods to boost power for rare variants. We analyzed all rare variants in our sample (defined as any variant with MAF ≤ 0.01) using the program rvTDT [15]. rvTDT collapsed rare variants into a single marker that corresponds to a gene or intergenic region, which are then analyzed using several different burden style tests which include: Collapsed Multivariate Method (CMC) [16]; Burden of Rare Variants (BRV); Weighted Sum Statistic (WSS) (Madsen and Browning, 2009); and Variable Threshold (VT) [17].

## 3. Results

### 3.1. De Novo Variant Analysis

We discovered 34 *de novo* variants in our sNCS trios (Table S2) and 11 *de novo* in our mNCS trios (Table S3), although none were in coding regions. Eight variants were found in intergenic regions and 26 in the intronic regions for sNCS whereas the corresponding totals for mNCS were six and five. Of interest in the sNCS trio *de novo* variants were: T/-deletion at rs1430021850 in one trio (MAF = 0.00), and an A/-deletion at rs374617290 in 7 trios (MAF = 0.014). Both variants are in candidate cis-Regulatory Elements identified by ENCODE (www.screen.encodeproject.org, accessed on 14 February 2022). For mNCS, two *de novo* variants were found in DNase I hyperactivity regions, indicative of a regulatory region, and found in one trio each: rs116959857 (MAF = 0.026) and an insertion at chr20:55791546 (MAF = 0.00). The genotypes of each *de novo* variant identified were zeroed out just for the trios involved and only for the variant involved prior to running TDT and rv-TDT.

### 3.2. Coding Rare Variants

No coding variants considered as damaging by Sift [18], Polyphen2 [19], or PROVEAN [20] were identified among the sNCS trios (Table 1). For the mNCS trios, six coding variants classified as damaging were identified, of which one, a p.Cys37Trp missense variant in *RTFDC1,* was a novel previously unreported variant. Five of the six variants were only found in one child-parent pair each (Table 1). A p.Val58Phe missense variant in the *ZBP1* gene, rs34917164, found in three mNCS child-parent trios, was classified as likely benign by ClinVar (https://www.ncbi.nlm.nih.gov/clinvar/, AccessionID: VCV000779046.1, accessed on 14 February 2022). Although classified as potentially pathogenic by SIFT, PolyPhen2, and PROVEAN, and having a MAF consistent with pathogenic variants, rs34917164 is presently classified as a variant of unknown significance (VUS).

### 3.3. TDT sNCS

**Figure 1:**
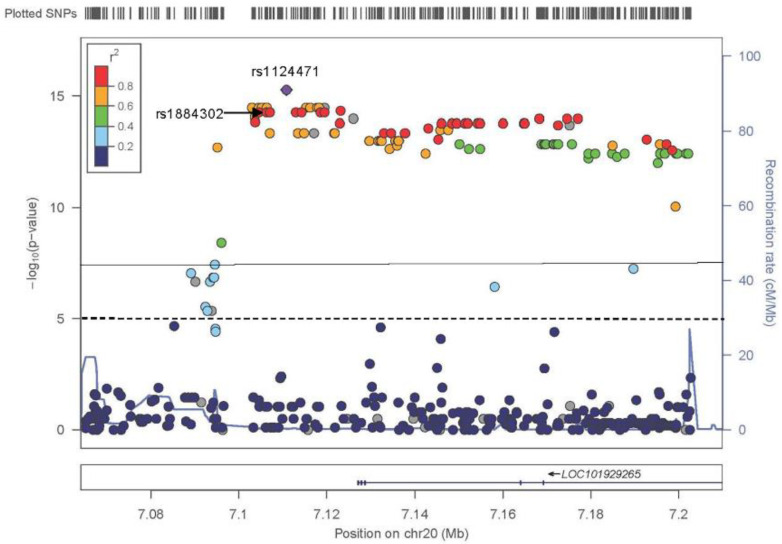
The results of the TDT using the sagittal suture phenotype from chromosome 20 using all 83 trios. The straight line at 7.3 and dashed line at 5 represents the genome-wide significant and genome-wide suggestive thresholds of 5.0 × 10^−8^ and 1.0 × 10^−5^ respectively. The LD (*r^2^*) between the top associated SNV rs1124471 (*p* = 5.27 × 10^−16^) and other SNVs are indicated. A grey dot indicates lack of LD between variant and top associated SNP. The location of rs1884302, the most significantly associated SNV from the sNCS GWAS, is indicated with an arrow. GWAS, genome-wide association study; LD, linkage disequilibrium; sNCS, sagittal nonsyndromic craniosynostosis; SNV, single nucleotide variant; TDT, transmission disequilibrium test.

The TDT results for sNCS yielded 118 significant results (*p* < 1 × 10^−5^), of which 116 were located on chromosome 20p12.3 in a region of approximately 107,000 bp (Figure 1). All variants were located either in the intergenic region between *BMP2* and the noncoding RNA gene *LINC01428* or in the intronic regions of *LOC101929265*. The most significant variant was rs1124471 (*p* = 5.27 × 10^−16^), and an additional 100 variants had a *p*-value ≤ 1.00 × 10^−10^. Table S4 lists all variants with a *p*-value < 0.0001. No variants on chromosome 3 reached the significance threshold (*p* < 1 × 10^−5^) (Figure S1), and only two variants on chromosome 7 reached this threshold (Figure S2, Table S4).

NCS is a complex phenotype characterized by locus heterogeneity, thus it is a reasonable possibility that some of these families are driven by the chromosome 7 signal, others by the chromosome 20 signal, which may still be present, even after the selection of only those trios with a chromosome 20 risk allele. This was the point of the leave-one-out analysis. This selection of trios with the chromosome 20 risk allele would explain why the leave-one-out analysis did not significantly alter the signal on chromosome 20, nor did it significantly increase the signals on chromosomes 3 or 7 for the sNCS trios.

We did identify a long, linked haplotype across the 20q12.3 region (Figure 1). It is possible that along this haplotype there is one causal variant in strong LD with other variants or that a cumulative effect of multiple variants was responsible for this signal.

Our rvTDT analysis found that the intergenic region between *BMP2* and *LOC101929265* was significant in both the haplotype adaptive versions of the CMC and VT methods (*p* = 0.02 and 0.01, respectively). This region was the only significant finding in the rare variant analysis of the sNCS data and suggests that rare variants are contributing to the disease risk for sNCS in the *BMP2*-*LOC101929265* intergenic region. This agrees with our standard TDT results, which identified a highly significant linked haplotype, extending from the *BMP2*-*LOC101929265* intergenic region into the *LOC101929265* gene proper. However, because all associated variants have similar *p*-values and exist along the same haplotype, it can often be difficult to determine the true causal variant. Annotation can be a useful filter in determining causality, although all variants identified were in noncoding regions. The most notable variant was rs147839746, (*p*-value 4.72 × 10^−13^) a four base pair deletion (AAAG) in the intergenic region between *BMP2* and *LOC101929265* (Table 2), with a MAF of 0.2179 in gnomAD. Although its *p*-value (4.72 × 10^−13^) was not quite as significant as some of the most significant variants, this is primarily because 10 trios did not have genotype information at this deletion. Of interest is the fact that rs147839746 is predicted to be a TFBS with a 99.5% probability by RegulomeDB and that TFBS contains motifs for three discrete transcription factors—*EWSR1*, *FOXP1*, and *TFAP2E*.

As the rvTDT analysis indicated the possibility of rare variants being partially responsible for the signal in the intergenic region between *BMP2* and *LOC101929265*, we annotated all rare variants in that region. Thirty-two rare variants had a greater than 50% probability of being a TFBS, including four variants that had a greater than 75% probability of being a TFBS. The most compelling of these was rs6054725, which has a 93% probability of being a TFBS and is a motif for *DXB1.*

### 3.4. TDT mNCS

**Figure 2:**
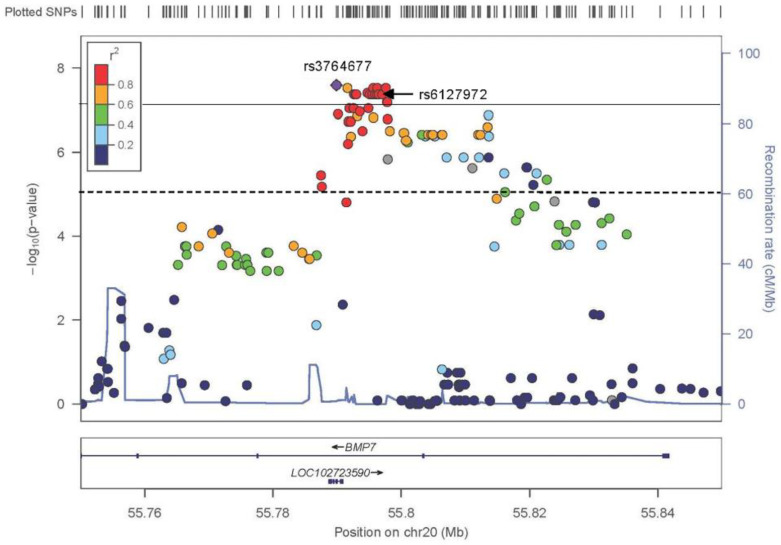
The results of the TDT for the metopic synostosis when all 80 trios are included in the analysis. Here, the dotted line represents the genome-wide suggestive threshold of 1.00 × 10^−5^, andthe straight line represents the genome-wide significant threshold of 5 × 10^−8^. The LD (*r^2^*) between the top associated SNV rs3764677 (*p* = 2.55 × 10^−8^) and other SNVs are indicated. The location of rs6127972, the most significantly associated SNV from the mNCS GWAS, is indicated with an arrow. GWAS, genome-wide association study; LD, linkage disequilibrium; mNCS, metopic nonsyndromic craniosynostosis; SNV, single nucleotide variant; TDT, transmission disequilibrium test.

No variant on chromosome 7 reached statistical significance (*p* < 1 × 10^−5^) (Figure S3). The most significant association identified on chromosome 20 was for rs3764677, an ncRNA_exonic variant in *BMP7-AS1* (*p* = 2.55 × 10^−8^) (Figure 2). Table S5 lists all variants with a *p* < 0.0001.

### 3.5. TDT mNCS Chromosome 7 Trios

**Figure 3:**
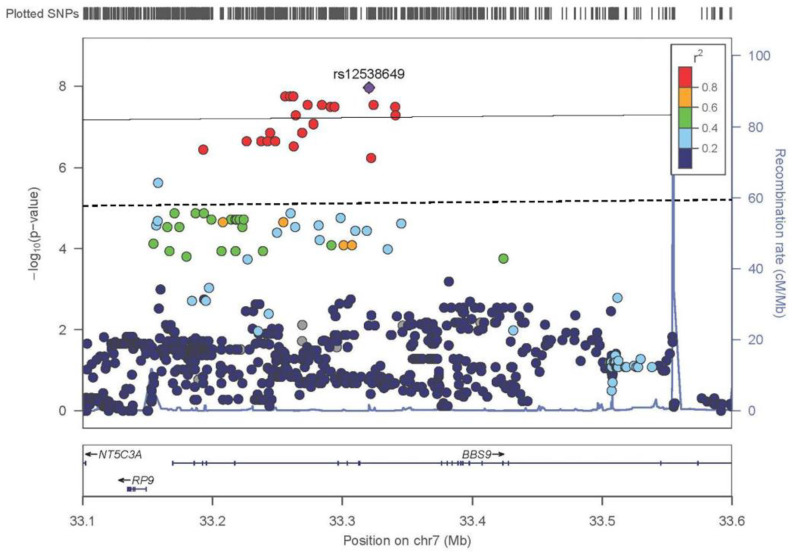
The results of the TDT for the metopic synostosis when only the 60 most informative trios are included. Here, the lines represent the genome-wide significant threshold of 5.00 × 10^−8^ and the genome-wide suggestive threshold of 1.00 × 10^−5^. The LD (*r^2^*) between the most significantly associated SNV rs12538649 (*p* = 1.09 × 10^−8^) and other SNVs are indicated. LD, linkage disequilibrium; SNV, single nucleotide variant; TDT, transmission disequilibrium test.

Using a leave-one-out analysis, we were able to separate the mNCS trios into groups that were responsible for the signal on either chromosome 7 or 20. The signal on chromosome 7 was driven by 60 trios. When TDT was performed on these trios, there were 61 variants that had a *p <* 1.0 × 10^−5^ with 11 variants reaching the standard genome-wide significance threshold (*p* < 5.0 × 10^−8^) (Figure 3, Table S6). All variants were either in the introns of *BBS9* or the intergenic region between *BBS9* and *RP9P* ranging from 33,156,578 –33,341,081 base pairs. The most significant variant was an intronic *BBS9* SNV, rs12538649 (*p* = 1.09 × 10^−8^, odds ratio [OR] = 8).

Our rvTDT analysis did not find either *BBS9* or the *RP9P-BBS9* intergenic region to be significant, meaning the signal was not being driven by rare variants, but instead by the common variants identified in the TDT analysis. It is likely these variants have an additive effect because they are in noncoding regions and may be regulatory in nature. From the variants determined to have a potential causal effect, as defined by RegulomeDB, the strongest association was to rs4723276, located in an intron of *BBS9*, with a *p* = 1.78 × 10^−8^, an OR = 7.83, and a MAF of 0.30. This variant has a 100% probability of being in a TFBS for the transcription factors *HOXB8* and *MSX1* (Table 3).

### 3.6. TDT mNCS Chromosome 20 Trios

**Figure 4:**
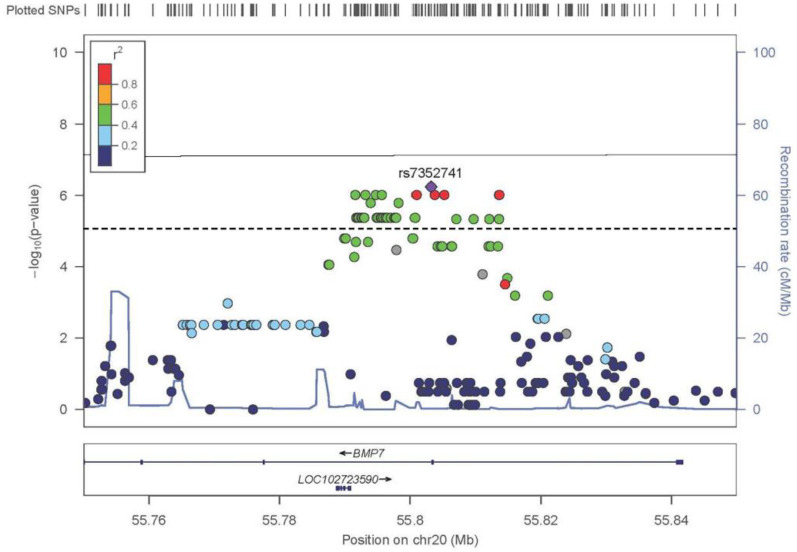
The results of the TDT for the metopic synostosis when only the 20 most informative trios are included. Here, the line represents the genome-wide suggestive threshold of 1.00 × 10^−5^ and the genome-wide significant threshold of 5.00 × 10^−8^. The LD (*r^2^*) between the most significantly associated SNV rs7352741 (*p* = 5.73 × 10^−7^) and other SNVs are indicated. LD, linkage disequilibrium; SNV, single nucleotide variant; TDT, transmission disequilibrium test.

The signal on chromosome 20 was driven by 20 trios. TDT analysis resulted in a distinct, sharp peak in the *BMP7* gene of approximately 22,800 bp (Figure 4). Forty-five variants were identified as significant (*p* ≤ 1.0 × 10^−5^) with each variant located in intronic regions of *BMP7*. The strongest association was to rs7352741 (*p =* 5.73 × 10^−7^). Table S7 lists all variants with a *p* < 0.0001.

Unlike the signal on chromosome 7 where the association was caused by the minor allele being transmitted to the affected child, the signal on chromosome 20 was reversed. Here, the association was driven by the over-transmission of the major alleles. This may be indicative of the minor allele having a protective effect on mNCS, with the major allele being the risk variant.

**Figure 5:**
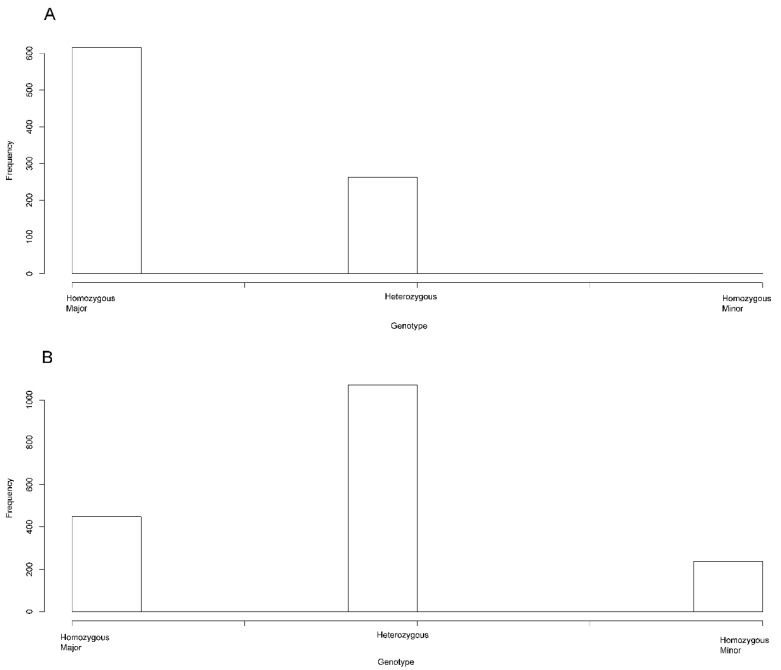
Histograms of the children (**A**) and parents (**B**) of the genotypes of the genome-wide significant chromosome 20 variants in 20 trios. The frequencies of the homozygous major, heterozygous, and homozygous minor genotypes for the 44 associated variants (rs6127972 not included) are displayed as bar graphs.

It is likely that the major alleles also have an additive effect. The probands were greatly enriched for the major alleles when compared to their parents for the 45 significantly associated (*p* ≤ 1.0 × 10^−5^) variants. Although our trios were selected based on their rs6127972 genotype (46 T/G and 34 T/T), the finding that the other associated variants were also the major alleles was not an expected outcome. Leaving the rs6127972 variant out for which our trios were selected, the genotype percentages of the parental generation were 25.5% homozygous major, 61.0% heterozygous, and 13.5% homozygous minor. By contrast, the children were 70.0% homozygous major, 30.8% heterozygous, and only 0.001% homozygous minor (Figure 5). There was a large inflation of the major alleles in the probands for these variants in *BMP7*, suggesting an additive effect of the major alleles mentioned above.

There are several good candidates among that group of associated common variants identified in *BMP7* (Table 4). The most interesting of these variants was rs35111023, which has a 93.10% probability of being a TFBS to *FOXA1* and *FOXA2*.

### 3.7. Chromosome 7 sNCS and mNCS

One unusual finding from our sNCS GWAS and mNCS GWAS was the fact that rs10262453 had an opposite effect depending on the suture involved. For sNCS, the A allele was over-transmitted, whereas for mNCS the C allele was over-transmitted. None of the trios sequenced for this study were selected based on their rs10262453 genotype, and the *p*-values for this variant were found to be much less significant than in the GWAS results for each phenotype, most likely due to the smaller sample size: *p* = 3.18 × 10^−5^ vs. *p* = 1.61 × 10^−10^ in the sNCS GWAS; *p* = 0.0011 vs. 4.48 × 10^−5^ in the mNCS GWAS. Looking more closely at this region on chromosome 7 across both phenotypes, we identified one variant, rs12538649, with a *p*-value < 0.0005 in both the sNCS and mNCS TDT analysis that also had an opposite effect: G allele (reference allele in gnomAD NFE with a MAF = 0.70) was over-transmitted in sNCS (*p* = 0.0001), whereas the C allele was over-transmitted in mNCS (*p* = 0.0003). This variant, rs12538649, had a relatively high rank score (0.6855) in RegulomeDB and was in a TFBS for *SLC20A1*.

## 4. Discussion

We had previously identified GWAS TDT associations on chromosomes 3, 7 and 20 in sNCS and chromosomes 7 and 20 in mNCS. Using targeted sequencing around these signals on our most informative families, we were able to narrow our previous signal to a highly significant region for sNCS in the introns and intergenic region around the ncRNA gene *LOC101929265*. We also identified two significant regions for mNCS, in the intronic/intergenic region around *BBS9* on chromosome 7 and in the introns of *BMP7*. These associated variants were found to be in noncoding regions and likely affect gene regulation. We were also able to use annotation to identify good candidates for potential causal variants within these signals. However, without the ability to perform any functional studies on these variants, their clinical value remains uncertain.

Of interest is the fact that the intronic region on *BBS9* seems to play a role in both mNCS and sNCS, although different alleles are involved. It would suggest that regulatory activity at this locus, in addition to the regulatory activity at the *BMP7* region for mNCS and at *BMP2* for sNCS, is involved in the phenotype. Several TFBSs were found in these regions, many of them plausible candidates which could result in a midline NCS phenotype.

Most intriguing perhaps is that several of the best candidate variants for sNCS were also involved in neural crest regulation. rs147839746 (*p* = 4.72 × 10^−13^) is a four-base pair deletion (AAAG) in the intergenic region between *BMP2* and *LOC101929265* (Table S4). It is strongly predicted (99.5%) to be a TFBS containing motifs for multiple transcription factors, including *FOXP1* and *TFAP2E*. *FOXP1* has been found to regulate jaw development [21], whereas *TFAP2E*, like both *MSX1* and *FOXA2*, has been shown to regulate the neural crest in frogs [22]. The *TFAP2* family of transcription factors, in general, is known to regulate craniofacial development [23]. Rare variant TDT analysis also suggested the presence of rare variants contributing to genetic risk in sNCS; the best candidate rare variant is rs6054725, a TFBS for *DXB1*. *DXB1* has been linked to craniofacial development [24].

Several potential TFBSs are excellent candidate variants for mNCS. Perhaps the most important variant was rs4723276, an intronic *BBS9* variant that was found to be genome-wide significant for mNCS (*p* = 1.78 × 10^−8^) when using the chromosome 7 mNCS families. rs4723276 is known to be a TFBS for *MSX1*, which is particularly intriguing with respect to CS. *MSX1* is known to play a key role in craniofacial patterning [25]. Suppression of *MSX1* in mice results in craniofacial abnormalities [26] and is involved in the transformation of neural crest cells into cartilage and bone [27].

Another finding of significance was that rs35111023 (*p* = 1.62 × 10^−6^), associated with the chromosome 20 mNCS families, was also a likely TFBS for a gene expressed in the neural crest, *FOXA2*. *FOXA2* is known to interact with *FOXG1* to regulate the facial neural crest, a pluripotent embryonic structure that gives rise to craniofacial features [28]. *FOXA2* is also known to stimulate bone morphogenesis [29]; thus, we have identified two likely TFBSs associated with mNCS that bind with genes known to affect craniofacial development.

We observed that the signals on chromosomes 7 and 20 in mNCS have multiple significant variants that are likely part of a linked haplotype. This means that there are multiple potential candidates for causality, and we attempted to provide our best estimate of causality based on annotation and biological function. We also observed that complex diseases, such as craniosynostosis, are almost certain to exhibit locus heterogeneity. Indeed, we observed locus heterogeneity in this study; some mNCS trios were more strongly associated with chromosome 7 and others with chromosome 20. It is, therefore, likely that different families have different associated variants (possibly within the same gene or regulatory region), and that an additive, polygenic effect and multiple variants across these regions are responsible for midline NCS. Of special interest was the fact that mNCS children are enriched for the homozygous major genotypes in *BMP7*. Given the reference would be prevalent in most individuals not affected with mNCS, which is a rare condition (1 in 10,000), this suggests that variants at other regulatory regions, or environmental factors, such as a fetal head constraint [30], might be necessary to produce the phenotype. The fact that both mNCS and sNCS have associations to the same region on chromosome 7 implies that a regulatory region at this locus contributes to both phenotypes, either directly or through interaction with a region involved with the regulation of *BMP2* and *BMP7*.

With only approximately 80 trios for each phenotype, our study was underpowered to identify rare variants, even when using the burden style test of rvTDT. This may account for the fact that we only had a single significant region in the rvTDT analysis—the intergenic region between *BMP2* and *LOC101929265*. Further sequencing using long read approaches is planned for this NCS study sample, which will likely allow for better calling and subsequent analysis of structural variants.

For the mNCS trios, six coding variants predicted as damaging were identified, of which one, a p.Cys37Trp missense variant in *RTFDC1,* was a previously unreported variant. Four of these variants were only found in one child-parent pair (Table 1). Homozygous and compound heterozygous pathogenic variants in *BMPER* (BMP binding endothelial regulator) cause diaphanospondylodysostosis and ischiospinal dysostosis, likely variable manifestations of the same skeletal dysplasia [31]. These patients present with reduced ossification of the skull and the axial skeleton, and occasionally with visceral abnormalities. Many of these genes were predicted to be TFBS and loss of function pathogenic variants in several of these TFBS target genes (*ALX4*, *NELL1*, *MSX2*, *RUNX2*) result in delayed skeletal ossification, whereas gain of function (GOF) variants in the same genes result in accelerated ossification and craniosynostosis [5]. A potential GOF effect of the BMPER p.Arg555Trp variant may be plausible, given the same variant was been previously identified in two out of 40 patients with sNCS [32]. *ZBP1* is highly expressed in osteoblasts and was found to be upregulated during osteogenic differentiation of mesenchymal stem cells (MSCs). It is a Wnt/β-catenin target that is required for β-catenin translocation into nuclei [33]. *ZBP1* depletion inhibits the expression of *RUNX2* and *AXIN2,* both genes associated with craniosynostosis. A p.Val58Phe missense variant in the *ZBP1* gene was found in three mNCS probands; these variants were classified as likely benign by ClinVar (https://www.ncbi.nlm.nih.gov/clinvar/, accessed on 14 February 2022) but as potentially pathogenic by SIFT, PolyPhen2, and PROVEAN.

The trios targeted for sequencing were selected because they were informative at the most significantly associated variant (rs1884302 for sNCS and rs6127972 for mNCS) in our GWASs. As such, these families were enriched at these SNVs and most additional SNVs genotyped in the region will be in LD with these previously associated SNVs. In theory, this approach could lead to the results of our study being biased; however, the objective of a targeted sequencing or fine-mapping study is to ask which variants in this targeted region have the highest potential for causality by performing the analyses on a much denser marker map.

Our previous functional studies found that a single allele change at rs1884302, the most significantly associated variant in our sNCS GWAS, affected the regulatory pattern observed in transgenic zebrafish [34]. Despite large numbers of noncoding variants having similar *p*-values, we were able to use annotation for TFBS, as well as biological significance, to identify the best potential variants for causality, including rs147839746 for sNCS and rs4723276 and rs35111023 for mNCS. We plan on performing additional in vitro and in vivo functional analyses to validate these findings, in an effort to better understand the regulatory processes which result in midline NCS.

In summary, with our denser marker map, we were able to narrow the association signals down to potential causal variants. Although we did not identify any coding variants or *de novo* pathogenic variants in the genes located in the associated regions; noncoding variants with predicted regulatory roles as TFBS were identified. Several of these affect genes that regulate craniofacial development and neural crest formation, including FOXA2, FOXG1, and MSX1 in mNCS and *TFAP2E* and FOXP1 in sNCS. We also identified *BMPER* and *ZBP1* as candidate genes for mNCS. Further, the association of midline NCS to two separate loci on chromosomes 7 and 20 underscores the genetic heterogeneity of this phenotype and the difficulty in resolving potential causality. Our findings suggest that NCS is a complex clinically and a genetically heterogeneous birth defect that may be caused by dysregulation of osteogenic developmental pathways by noncoding variation. Whole genome sequencing of large and clinically homogeneous NCS study samples, along with functional studies of the noncoding variants discussed here, are the next logical steps toward a better understanding of the etiology and pathogenesis of NCS.

## Figures and Tables

**Table 1:** Missense variants in 83 sNCS and 80 mNCS trios.

Gene	Variant	Chr:Bp	Rs ID	Sift	PolyPhen2	Provean	MAF	Suture	P-O Pairs
*DLG1*	c.521G>C (p.Pro174Arg)	3:196,888,572	rs375133714	0.026	0.15	−1.27	0.00010	sagittal	1
*BBS9*	c.1760G>A (p.Arg587Gln)	7:33,407,445	rs149042169	0.68	0	−0.08	0.00013	sagittal	1
*BMPER*	c.1663C>T (p.Arg555Trp)	7: 34,125,622	rs10249320	0.01	1	−4.55	0.00815	metopic	1
*CASS4*	c.110T>C (p.Ile37Thr)	20:55,012,293	rs958032346	0	0.96	−4.32	NA	metopic	1
*RTFDC1*	c.109T>G (p.Cys37Trp)	20:55,048,398	unknown	0	1	−10.63	NA	metopic	1
*FAM209A*	c.165G>T (p.Trp55Cys)	20:55,100,029	rs139125762	0	1	−12.21	0.00022	metopic	1
*FAM209A*	c.409A>G (p.Met137Val)	20:55,101,019	rs149932128	0.14	0	−2.33	0.00303	metopic	1
*SPO11*	c.547A>G (p.Arg183Gly)	20:55,909,842	rs137997364	0.39	0	−2.3	0.00079	metopic	2
*RBM38*	c.478G>A (p.Ala160Thr)	20:55,982,660	rs766651552	0.4	0	−0.54	0.00003	metopic	1
*RBM38*	c.661G>A (p.Ala221Thr)	20:55,982,843	rs201744631	0.2	0.02	−1.48	0.00088	metopic	1
*CTCFL*	c.1159C>G (p.Ala387Pro)	20:56,093,714	rs536304823	NA	0.4	NA	0.00106	metopic	1
*CTCFL*	c.520C>T (p.Ala174Thr)	20:56,098,742	rs761924648	0.41	0.16	−1.11	0.00001	metopic	1
*PCK1*	c.410G>A (p.Arg137His)	20:56,137,755	rs150560473	0	1	−3.16	0.00021	metopic	1
*PCK1*	c.413C>T (p.Thr138Ile)	20:56,137,758	rs28359542	0.06	0.98	−3.47	0.00371	metopic	2
*ZBP1*	c.172C>A (p.Val58Phe)	20: 56,191,387	rs34917164	0	1	−3.85	0.00783	metopic	3
*C20orf85*	c.60T>G (p.Asp20Glu)	20: 56,726,080	rs200557259	0.01	0.03	−2.74	0.00053	metopic	1

Bold text indicates variants considered as damaging per Sift, PolyPhen2 and PROVEAN. Chr:bp: chromosome, base pairs position GRCh37; Sift score 0.0–0.05 damaging; PolyPhen2 score 0.85–1 damaging; PROVEAN ≤ −2.5 damaging; MAF, minor allele frequency gnomAD non-Finnish European; P-O pairs, number of child-parent pairs with an alternate allele.

**Table 2:** Potential causal variants for sNCS in chromosome 20 target region.

CHR	Variant	BP	T	U	OR	*p*-Value	Function	Type	Gene	MAF	TFBS Probability
20	rs147839746	7117108	80	11	7.273	4.72 × 10^−13^	Intergenic	DEL	BMP2; LOC101929265	0.2178	0.9950
20	rs3028890	7126056	90	13	6.923	3.27 × 10^−14^	Intergenic	DEL	LOC101929265	0.3203	0.78026
20	rs6054770	7131752	83	13	6.385	9.04 × 10^−13^	ncRNA_intronic	SNV	LOC101929265	0.2604	0.64575
20	rs2207588	7147515	81	12	6.75	8.37 × 10^−13^	ncRNA_intronic	SNV	LOC101929265	0.2617	0.66931
20	rs71330228	7175140	10	82	0.122	6.07 × 10^−14^	ncRNA_intronic	DEL	LOC101929265	0.4127	0.77072
20	rs71330230	7201677	76	12	6.333	8.95 × 10^−12^	ncRNA_intronic	SNV	LOC101929265	0.1721	0.995

BP, base pair position (GRCh37/hg19); CHR, chromosome; MAF, minor allele frequency gnomAD non-Finnish European; OR, odds ratio; T, transmitted; TFBS, transcription factor binding site; Type, type of variant; U, untransmitted.

**Table 3:** Selected potential causal variants in chromosome 7 and 20 target regions from mNCS leave-one-out analysis.

CHR	Variant	BP	T	U	OR	*p*-Value	Function	Type	Gene	MAF	TFBS Probability
7	rs11763098	33,158,020	14	55	0.2545	7.98 × 10^−7^	Intergenic	SNV	RP9; BBS9	0.4640	0.58955
7	rs2006387	33,214,278	15	53	0.2830	4.06 × 10^−6^	Intronic	SNV	BBS9	0.4792	0.58955
7	rs10262453	33,256,039	58	10	5.8	5.86 × 10^−9^	Intronic	SNV	BBS9	0.2989	0.58955
7	rs4723276	33,259,793	58	10	5.8	5.86 × 10^−9^	Intronic	SNV	BBS9	0.3001	1
7	rs12538649	33,320,648	59	11	5.364	9.63 × 10^−9^	Intronic	SNV	BBS9	0.2962	0.6855
7	rs1978333	33,322,230	52	11	4.727	2.40 × 10^−7^	Intronic	SNV	BBS9	0.2945	0.60906
20	rs3067084	55,794,451	0	24	0	9.63 × 10^−7^	Intronic	DEL	BMP7	0.3822	0.66633
20	rs35111023	55,793,972	0	23	0	1.62 × 10^−6^	Intronic	SNV	BMP7	0.4132	0.93104
20	rs182795	55,792,907	1	24	0.04167	4.23 × 10^−6^	Intronic	SNV	BMP7	0.4165	0.64591
20	rs1475000	55,792,997	1	24	0.04167	4.23 × 10^−6^	Intronic	SNV	BMP7	0.4145	0.82852
20	rs172982	55,795,918	1	24	0.04167	4.23 × 10^−6^	Intronic	SNV	BMP7	0.4141	0.71939
20	rs56404749	55,797,901	1	20	0.05	3.38 × 10^−5^	Intronic	DEL	BMP7	0.455	0.87
20	rs2180780	55,800,721	1	24	0.04167	4.23 × 10^−6^	Intronic	SNV	BMP7	0.4541	0.66784
20	rs35420824	55,813,556	0	21	0	4.59 × 10^−6^	Intronic	SNV	BMP7	0.3167	0.70497

BP, base pair position (GRCh37/hg19); CHR, chromosome; MAF, minor allele frequency gnomAD non-Finnish European; OR, odds ratio; T, transmitted; TFBS, transcription factor binding site; Type, type of variant; U, untransmitted.

**Table 4:** Selected potential causal variants in chromosome 7 and 20 target regions from mNCS leave-one-out analysis.

CHR	Variant	BP	T	U	OR	*p*-Value	Function	Type	Gene	MAF	TFBS Probability
7	rs11763098	33,158,020	14	55	0.2545	7.98 × 10^−7^	Intergenic	SNV	RP9; BBS9	0.4640	0.58955
7	rs2006387	33,214,278	15	53	0.2830	4.06 × 10^−6^	Intronic	SNV	BBS9	0.4792	0.58955
7	rs10262453	33,256,039	58	10	5.8	5.86 × 10^−9^	Intronic	SNV	BBS9	0.2989	0.58955
7	rs4723276	33,259,793	58	10	5.8	5.86 × 10^−9^	Intronic	SNV	BBS9	0.3001	1
7	rs12538649	33,320,648	59	11	5.364	9.63 × 10^−9^	Intronic	SNV	BBS9	0.2962	0.6855
7	rs1978333	33,322,230	52	11	4.727	2.40 × 10^−7^	Intronic	SNV	BBS9	0.2945	0.60906
20	rs3067084	55,794,451	0	24	0	9.63 × 10^−7^	Intronic	DEL	BMP7	0.3822	0.66633
20	rs35111023	55,793,972	0	23	0	1.62 × 10^−6^	Intronic	SNV	BMP7	0.4132	0.93104
20	rs182795	55,792,907	1	24	0.04167	4.23 × 10^−6^	Intronic	SNV	BMP7	0.4165	0.64591
20	rs1475000	55,792,997	1	24	0.04167	4.23 × 10^−6^	Intronic	SNV	BMP7	0.4145	0.82852
20	rs172982	55,795,918	1	24	0.04167	4.23 × 10^−6^	Intronic	SNV	BMP7	0.4141	0.71939
20	rs56404749	55,797,901	1	20	0.05	3.38 × 10^−5^	Intronic	DEL	BMP7	0.455	0.87
20	rs2180780	55,800,721	1	24	0.04167	4.23 × 10^−6^	Intronic	SNV	BMP7	0.4541	0.66784
20	rs35420824	55,813,556	0	21	0	4.59 × 10^−6^	Intronic	SNV	BMP7	0.3167	0.70497

BP, base pair position (GRCh37/hg19); CHR, chromosome; MAF, minor allele frequency gnomAD non-Finnish European; OR, odds ratio; T, transmitted; TFBS, transcription factor binding site; Type, type of variant; U, untransmitted.

## Data Availability

Not applicable.

## Supplementary Materials

The following supporting information can be downloaded at: https://www.mdpi.com/article/10.3390/genes13050816/s1, Table S1: Targeted regions sequenced for sNCS and mNCS trios; Table S2: *De novo* variants in sNCS trios; Table S3: *De novo* variants in mNCS trios; Table S4: sNCS TDT results for 83 trios, variants with *p* < 0.0001; Table S5: mNCS TDT results for 80 trios, variants with *p* < 0.0001; Table S6: mNCS TDT results for variants with *p* < 0.0001 using chromosome 7 trios (60 trios); Table S7: mNCS TDT results for variants with *p* < 0.0001 using chromosome 20 trios (20 trios); Figure S1: Chromosome 3 Sagittal Synostosis TDT Results using all Trios; Figure S2: Chromosome 7 Sagittal Synostosis TDT Results using all Trios; Figure S3: Chromosome 7 Metopic Synostosis TDT Results using all Trios.
