# Supplementary material for: Targeted Sequencing of Candidate Regions Associated with Sagittal and Metopic Nonsyndromic Craniosynostosis
Source: Genes (Basel). 2022 May 3;13(5):816. doi: 10.3390/genes13050816 (PMC9141801; doi:10.3390/genes13050816)

SUPPLEMENTAL FIGURES

Figure S1. Chromosome 3 Sagittal Synostosis TDT Results using all Trios. *P*-values (-log10) are plotted against the genomic position of each SNV on the chromosome 3 associated, with genes in the region depicted below. The LD (r^2) between the top associated SNV rs3773855 (P=1.42 x 10^{-5}) and other SNVs are indicated.

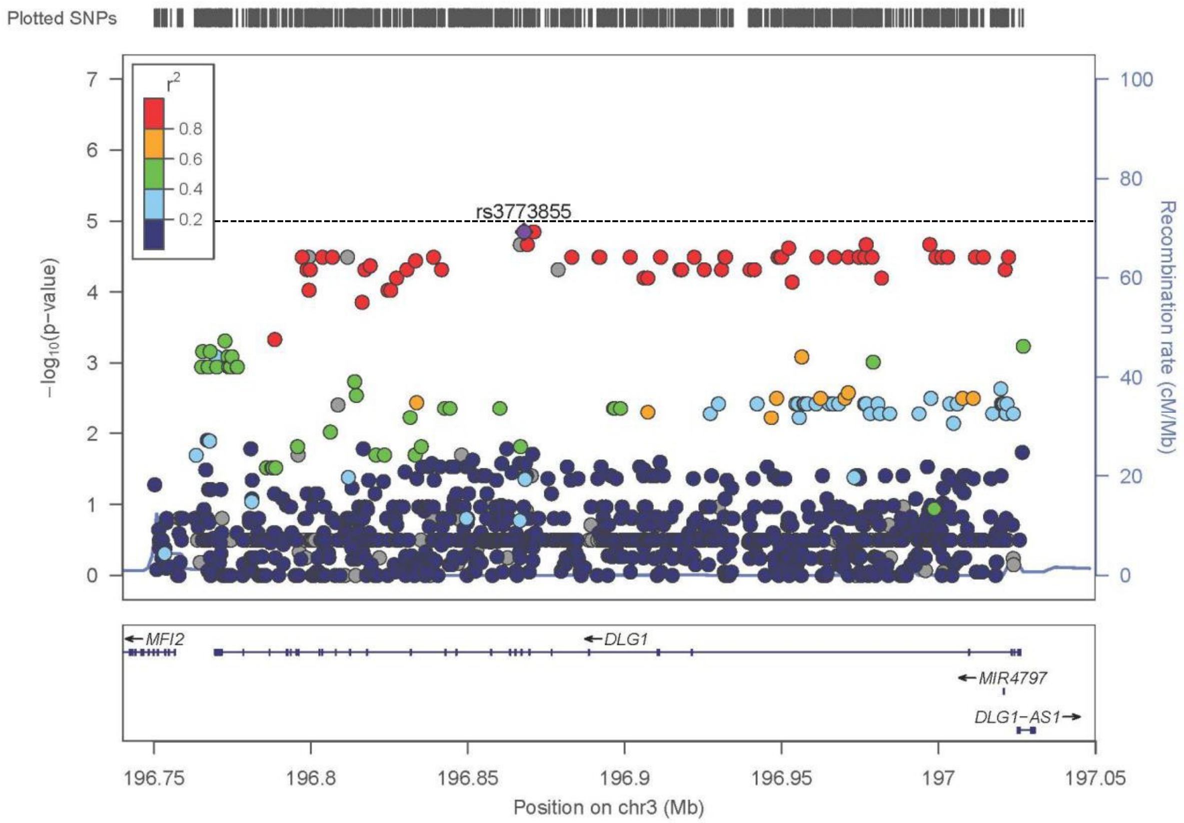

Figure S2. Chromosome 7 Sagittal Synostosis TDT Results using all Trios. *P*-values (-log10) are plotted against the genomic position of each SNV on the chromosome 7 associated, with genes in the region depicted below.The LD (r^2) between the top associated SNV rs1983511 (*P*=1.54 x 10^{-5}) and other SNVs are indicated. The dotted line represents the genome-wide suggestive threshold of 1 x 10^{-5}.

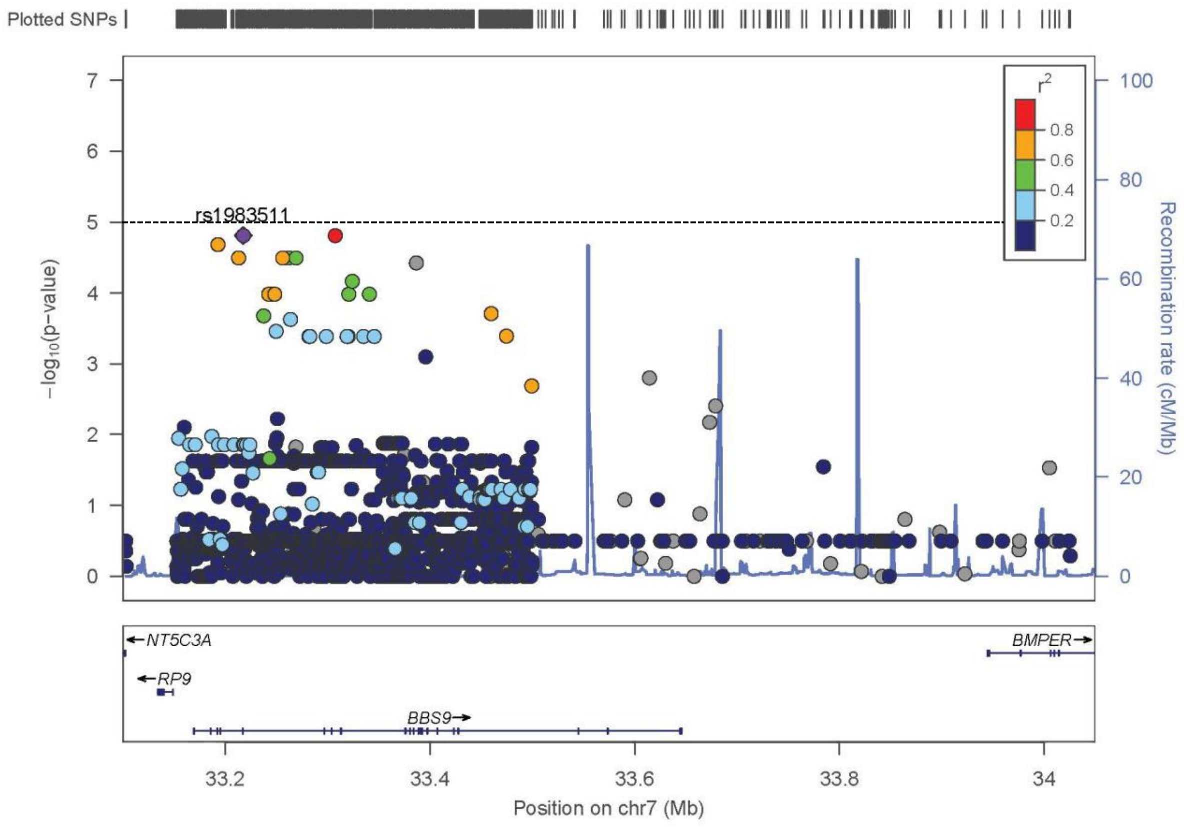

Figure S3. Chromosome 7 Metopic Synostosis TDT Results using all Trios. *P*-values (-log10) are plotted against the genomic position of each SNV on the chromosome 7 associated, with genes in the region depicted below. The LD (r^2) between the top associated SNV rs12538649 (*P*=0.00029) and other SNVs are indicated. Thedotted line represents the genome-wide suggestive threshold of 1 x 10^{-5}.

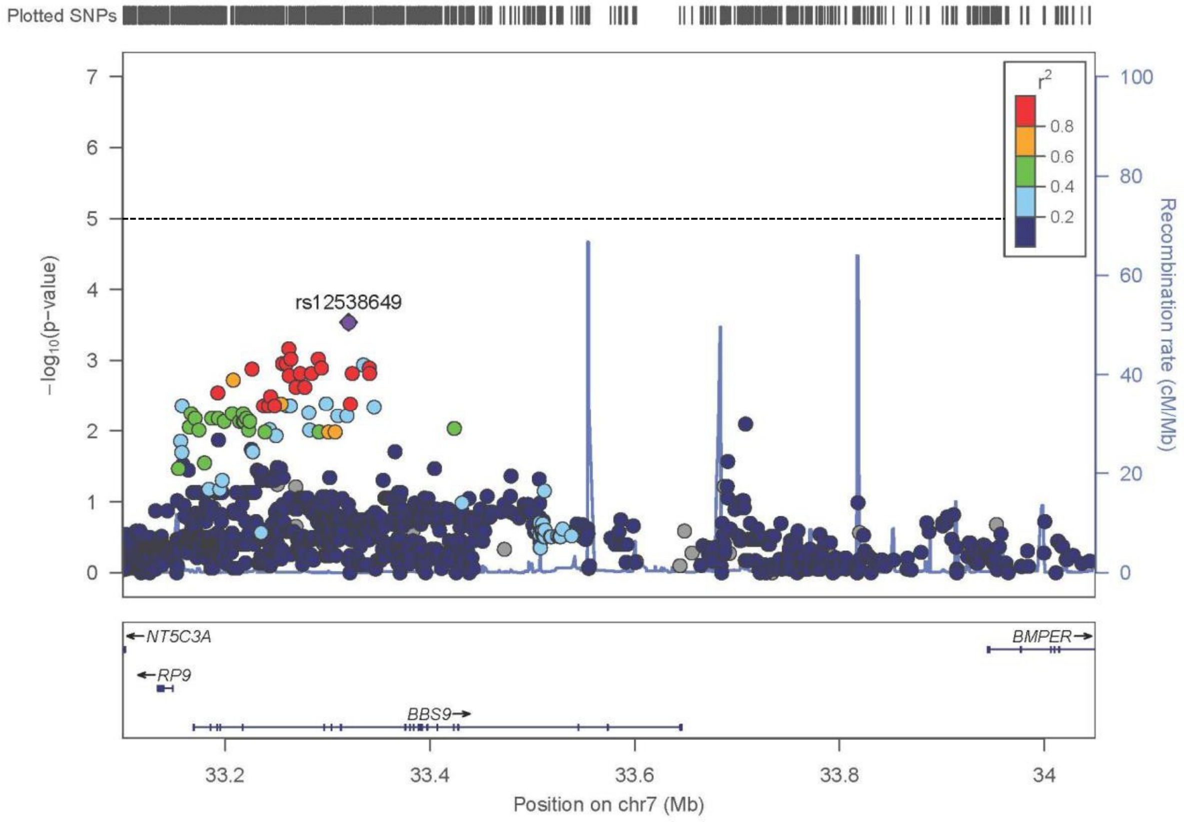

Supplement: Supplementary file 1 [file genes-13-00816-s001.zip › supplementary_figures.pdf]
